# Host responses to interspecific brood parasitism: a by-product of adaptations to conspecific parasitism?

**DOI:** 10.1186/1742-9994-11-34

**Published:** 2014-04-28

**Authors:** Peter Samas, Mark E Hauber, Phillip Cassey, Tomas Grim

**Affiliations:** 1 Department of Zoology and Laboratory of Ornithology, Palacký University, 17. listopadu 50, CZ-771 46 Olomouc, Czech Republic; 2 Department of Psychology, Hunter College and the Graduate Center of the City University of New York, 695 Park Avenue, New York, NY 10065, USA; 3 School of Earth and Environmental Sciences, University of Adelaide, Adelaide, SA 5005, Australia

**Keywords:** Coevolution, Collateral damage, Discrimination, Heterospecific brood parasitism, Intraspecific brood parasitism, Species introductions

## Abstract

**Background:**

Why have birds evolved the ability to reject eggs? Typically, foreign egg discrimination is interpreted as evidence that interspecific brood parasitism (IP) has selected for the host’s ability to recognize and eliminate foreign eggs. Fewer studies explore the alternative hypothesis that rejection of interspecific eggs is a by-product of host defenses, evolved against conspecific parasitism (CP). We performed a large scale study with replication across taxa (two congeneric *Turdus* thrushes), space (populations), time (breeding seasons), and treatments (three types of experimental eggs), using a consistent design of egg rejection experiments (n = 1057 nests; including controls), in areas with potential IP either present (Europe; native populations) or absent (New Zealand; introduced populations). These comparisons benefited from the known length of allopatry (one and a half centuries), with no gene flow between native and introduced populations, which is rarely available in host-parasite systems.

**Results:**

Hosts rejected CP at unusually high rates for passerines (up to 60%). CP rejection rates were higher in populations with higher conspecific breeding densities and no risks of IP, supporting the CP hypothesis. IP rejection rates did not covary geographically with IP risk, contradicting the IP hypothesis. High egg rejection rates were maintained in the relatively long-term isolation from IP despite non-trivial rejection costs and errors.

**Conclusions:**

These egg rejection patterns, combined with recent findings that these thrushes are currently unsuitable hosts of the obligate parasitic common cuckoo (*Cuculus canorus*), are in agreement with the hypothesis that the rejection of IP is a by-product of fine-tuned egg discrimination evolved due to CP. Our study highlights the importance of considering both IP and CP simultaneously as potential drivers in the evolution of egg discrimination, and illustrates how populations introduced to novel ecological contexts can provide critical insights into brood parasite-host coevolution.

## Introduction

Why do birds recognize their own eggs and reject foreign ones? This question has fascinated researchers for centuries [1]. Most previous studies concluded that birds discriminate foreign eggs as defence against interspecific brood parasites, e.g., common cuckoos (*Cuculus canorus*; hereafter: cuckoo) [2,3]. However, egg discrimination abilities are detected even in species that are not known to be impacted by interspecific parasites, including those that seem to be unsuitable cuckoo hosts [2].

Here, we investigate the potential causes of egg rejection in birds that are currently not impacted by interspecific brood parasitism, yet are known to be able to reject foreign eggs in the nest: *Turdus* thrushes [2]. Cuckoo parasitism was documented in all six species of thrushes that occur in Europe, and most often in our two study species, the European blackbird (*Turdus merula*; hereafter: blackbird) and the song thrush (*T. philomelos*) [4]. However, these parasitism rates were overall an order of magnitude lower than those in typical current or previous cuckoo hosts [4], casting doubts on the hypothesis that cuckoo parasitism was the selective force for egg rejection in European thrushes. Moreover, previous studies contradicted each other, classifying these thrushes as either suitable [2] or unsuitable [5] hosts for the cuckoo. A recent, large-scale study [6] suggested that thrushes are currently unsuitable hosts of cuckoos in Europe: under natural conditions, cuckoo chicks do not survive until fledging in thrush nests, which prevents long-term coevolution between cuckoos and thrushes; the alternative, but directly untestable interpretation is that cuckoo parasitism was prevalent in the distant past and these hosts have beaten it to cause the extinction of the thrush-race of cuckoos [7]. Regardless, cuckoo parasitism, even if currently unsuccessful for the cuckoo chick [6], is still costly for the host because of incubation costs of the foreign egg [8] and the egg eviction by the hatchling parasite [6]. But long-term existence of such costs from interspecific parasitism (IP) may be unlikely, given that cuckoos should evolve to avoid laying eggs in unsuitable hosts' nests, and thus cuckoos would impose small-to-no cost on those hosts. Therefore, a more plausible hypothesis might be that conspecific brood parasitism (CP) has selected for egg rejection [9,10].

Egg rejection in response to CP is tested considerably less often than host responses to IP [2,11-17]. The majority of brood parasitism studies considered only rejection of IP eggs (Figure one in [18]). Testing both IP and CP scenarios in the same study is crucial because rejection of IP eggs may theoretically be a by-product of host adaptations against CP; this “collateral damage” hypothesis was previously tested [2,9,11,16,19-24] but supported only in a single non-passerine, waterfowl system [24]. Here we provide the first empirical evidence for collateral damage in passerine birds.

IP and CP are not mutually exclusive as sources of selection for egg rejection, because both can operate in any particular host species [1,9]. If antagonistic interactions between both interspecific and conspecific parasites and their hosts converge to produce the same antiparasitic adaptation in host behaviors (egg rejection: [1]), then how can we differentiate between the two alternative functional explanations? Several types of concurrent experiments with consistent methodologies, but with alternative predictions, are required to test the two hypotheses (Table 1). These predictions are based on one of the cornerstones of evolutionary theory: “In the absence of these antagonistic interactions, hosts should be expected to lose their defenses either through genetic drift or natural selection.” [25], p. 162. General evolutionary theory predicts “evolutionary loss of useless structures” [26], p. 529. Therefore, behavioral and cognitive traits that are not positively selected, e.g., in allopatry with parasites, should decay because of mutation pressure [26], genetic drift [25,26], costs of maintenance of neural networks [27] and rejection costs and errors [28]. All of these factors independently and additively lead to decay of any organismal trait which does not have any current adaptive function. However, even without any genetic change, the same patterns are predicted from phenotypic plasticity: decreased realized or perceived parasitism pressure should lead to lower antiparasite responses [29]. Indeed, such patterns were often documented in cuckoo hosts (see below) but not in some hosts of North American brown-headed cowbirds (*Molothrus ater*) [30].

**Table 1:** Summary of contrasting predictions of conspecific parasitism (CP) and interspecific parasitism (IP) hypotheses and the results for the two focal host species in this study

**Response**	**CP**	**IP**	**Results of this study**	
	**(thrushes)**	**(cuckoo)**	**Blackbird**	**Song thrush**
Conspecific egg rejection	+	–	+	+
CP rejection rate	CZ<NZ	n.a.	CZ<NZ	CZ<NZ
CP rejection latency	CZ>NZ	n.a.	CZ<NZ	CZ~NZ
IP rejection rate	n.a.	CZ_S_>CZ_A_>NZ	CZ_S_<CZ_A_>NZ	CZ_S_~CZ_A_~NZ
IP rejection latency	n.a.	CZ_S_<CZ_A_<NZ	CZ_S_~CZ_A_~NZ	CZ_S_~CZ_A_~NZ

Populations of European blackbirds and song thrush, in the Czech Republic (CZ) are either sympatric (S) or micro-allopatric (A; as denoted by subscripts), populations in New Zealand (NZ) are all macro-allopatric with common cuckoos. CP predictions for Czech Republic vs. New Zealand populations are based on differences in the breeding densities of thrushes (higher in New Zealand for both species) and refer to host responses to conspecific eggs. IP predictions are based on sympatry vs. allopatry with cuckoos and refer to non-mimetic cuckoo-like model eggs. See Introduction for the rationale of predictions. n.a. = not applicable.

We took advantage of the known length of allopatry with IP in blackbirds and song thrush introduced to New Zealand where they live in isolation from common cuckoos; this allows for a powerful test for roles of IP and CP in the evolution of egg discrimination in these birds as already suggested by a pioneering study of [17] (see also [31,32], Methods, and Table 1). CP has been documented in both of these *Turdus* species, and in both their native (our study populations in Czech Republic) and introduced ranges ([13,33,34], our own observations), implying that the evolution of egg rejection in these taxa may have been due to parasitic egg laying by conspecifics. We tested following predictions:

(i) If CP selected for egg discrimination, then thrushes should be able to selectively reject foreign conspecific eggs. If IP selected for egg discrimination, then hosts should not reject conspecific eggs.

The evolution of fine-tuned egg discrimination is unnecessary in the absence of parasitic eggs that closely resemble those of hosts, e.g., from conspecific parasites or interspecific parasites with closely mimetic eggs [16,35]. This view is supported both by theory [36,37] and empirical data, i.e., the positive correlation between the match of cuckoo egg mimicry of host eggs, and the hosts’ egg discrimination abilities [3,38]. Crucially, most typical cuckoo hosts reject dissimilar eggs but accept conspecific eggs [2], except for taxa with the best mimicry of host eggs by the cuckoo (e.g., great reed warbler *Acrocephalus arundinaceus*: [39]). Known suitable cuckoo hosts/populations that do reject conspecific eggs are often currently avoided by cuckoos, but there is ample evidence for IP in historical and museum records [4] and, without exception, these species are/were parasitized by highly mimetic cuckoo eggs [11,16,19,40,41]. In contrast, no known cuckoo eggs are similar to *Turdus* eggs: cuckoo eggs are about half the size of thrush eggs [6] and do not closely resemble thrush eggs in either color or patterning [4]. Therefore, IP alone could not provide sufficient selection pressure on thrushes to evolve abilities to discriminate *conspecific* eggs [36].

(ii) If CP selected for egg discrimination, then egg rejection rates of conspecific natural eggs should be higher in populations with higher breeding densities.

Just as greater perceived risks of cuckoo parasitism (due to naturally higher cuckoo densities or their experimental presentations at host nests) increase host rejection of cuckoo eggs [2] and nest defense [2,42], perceived risks of conspecific parasitism (due to higher conspecific breeding densities) increase host rejection of conspecific eggs [43-45]. Our New Zealand study populations show consistent spatio-temporally higher densities (more than twice) than those of our European populations [32]. Historical data from the same general areas we studied suggest that both blackbird and song thrush breeding densities were twice as high or even higher in New Zealand than in Czech Republic at least a half century ago [46,47]. Therefore, New Zealand populations should reject conspecific eggs at higher rates than do the European populations. Our allopatric study populations [prediction (iii)] happen to be the ones with higher breeding densities; however, this is not a critical confound, and rather, a possible advantage because the CP and IP hypotheses make predictions which are opposite for these same populations and concern different types of experimental eggs (conspecific vs. cuckoo-like), and therefore provide a powerful test of our hypotheses (Table 1).

(iii) If CP selected for egg discrimination, then latency to egg rejection should be shorter in populations with higher breeding densities. Egg rejection may be quicker in denser host populations with higher perceived risks or realized costs of CP [45].

(iv) If IP selected for egg discrimination, then egg rejection rates of cuckoo-like models should decrease from sympatry, through micro-allopatry to macro-allopatry with cuckoos. Cuckoo hosts show high phenotypic plasticity and adjust their anti-parasite responses according to the perceived risk of parasitism [48]. Notably, egg rejection rates drop with a decline in the density of cuckoos within years [49] or decades [29,42,50]. Irrespective of the mechanism (rapid evolution [51], or phenotypic plasticity [29]), current cuckoo hosts typically show lower defenses in allopatry than in sympatry with cuckoos [2,48,49,52,53]. However, some allopatric host populations still reject experimental parasitism frequently [7]. For all such hosts there is ample evidence of frequent parasitism in the past and highly evolved cuckoo egg mimicry. There is no such evidence for any *Turdus* species. Egg rejection rates across host taxa and populations positively correlate with local IP rates [54]. In contrast, [6] found no evidence for such patterns in thrushes. However, [6] did not know the length of presumed sympatry/allopatry. Here, we studied not only populations in “micro-allopatry” (i.e., within Europe) where the length of allopatry cannot be known in principle but also populations in “macro-allopatry”, with known length of allopatry between distant regions with and without cuckoos (see Methods). In the New Zealand study populations, where local brood parasites do not use introduced species [17], the two thrush species have been isolated from common cuckoos for a period that is an order of magnitude longer (century and a half) than the duration of allopatry presumed in the studies conducted in Europe (see above), which provides the strongest available test of our hypotheses [17]. To our knowledge, only two study systems with known (and not estimated, [30,55]) length of allopatry with interspecific parasites were examined with consistent methods across different populations to date [17,20,35].

(v) If IP selected for egg discrimination then the latency to egg rejection of cuckoo-like models should increase from sympatry, through micro-allopatry to macro-allopatry with cuckoos. This is because the presence of adult cuckoos is known to increase the speed of host responsiveness to costly foreign eggs [53].

A survey of previous experimental work on blackbirds and song thrush in both sympatry [2,5,13,33,56,57] and allopatry [13,17,31] demonstrated consistently higher rejection rates of non-mimetic cuckoo-type eggs than conspecific-like model or real conspecific eggs. Generally, authors interpreted these patterns as a support for IP hypothesis. However, such patterns are equally consistent with CP hypothesis, which also predicts a graded response of higher rejection rates to increasingly dissimilar foreign egg phenotypes relative to their own eggs [58], irrespective of whether the foreign egg is that of a conspecific or a heterospecific (as predicted by [36]; see also [2,3,39,59]).

Published experimental methods, treatments, tools, and criteria have often varied between the host species, study sites, and areas with and without cuckoos in previous studies, preventing meaningful quantitative comparisons. Different authors used different model eggs with respect to material, size, and color ([2] vs. [17]), employed different criteria for assessing acceptance of alien eggs (6 days in [5] vs. 4 days in [56]), typically studied a single host population [33] and did not include experimental treatments with conspecific eggs between site types within the same study [13]. In the present work, we used (a) taxonomic replicates (two *Turdus* species), (b) geographical replicates (several populations within both allopatry and sympatry), (c) temporal replicates (different breeding seasons within each study site), and (d) treatment replicates (three types of experimental eggs) with (e) a large number of nests (1057 egg experiments; this is the largest sample size for egg experiments in a single study of brood parasitism to date). To address methodological constraints of previous studies, we employed consistent experimental approaches in all population (e.g., identical model eggs manufactured by one person) to generate quantitatively comparable results and strong tests of the alternative hypotheses.

## Results

Overall, we obtained information on host responses under the 6-day response criterion for 685 blackbird nests (402 blue, 106 spotted, 107 conspecific, 70 controls) and 372 song thrush nests (181 blue, 87 spotted, 61 conspecific, 43 controls).

### Nest desertion

In blackbirds, nests with experimental conspecific eggs were deserted statistically more often than control nests, when only statistically significant predictors were included in the model (Additional file 1: Appendix 1). But nest desertion was not a significant outcome of the experimental manipulation in blackbirds when other predictors were not taken into account. In song thrush, nest desertions did not statistically differ across treatments (Additional file 1: Appendix 1). Previous studies did not reach a consensus on whether nest desertion was a specific response to parasitism or not [6,57,60,61]. Therefore it remains unclear whether these statistical conclusions reflect a biological role of blackbird nest desertion in response to parasitism. According to our new statistical results here, we include desertion as a response only for conspecific treatment in blackbirds. We also, conservatively, present statistical models of egg rejection rates both including and excluding nest desertion for all treatments for both thrushes (Additional file 1: Appendix 2). This also makes our results quantitatively comparable to all previous studies, i.e., for future meta-analyses. All conclusions of the present study remain the same regardless of including and excluding nest desertion (Additional file 1: Appendices 1 and 2).

### Responses to experimental conspecific parasitism vs. breeding densities

**Figure 1:**
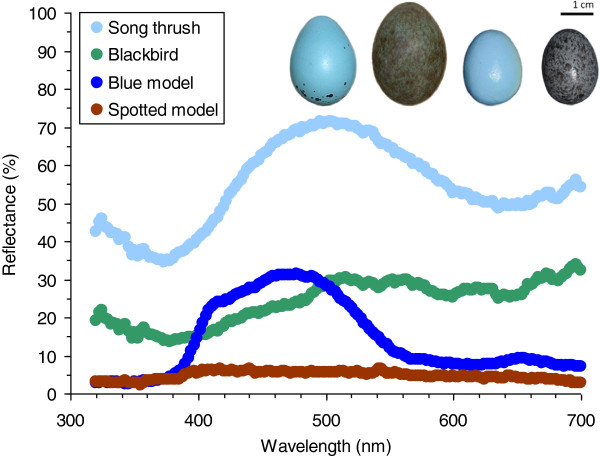
**Representative reflectance spectra (5 nm running means) of the eggs used in experiments.** Examples (from left to right) depict song thrush and blackbird natural eggs and blue (redstart) and spotted (meadow pipit) model eggs.

**Figure 2:**
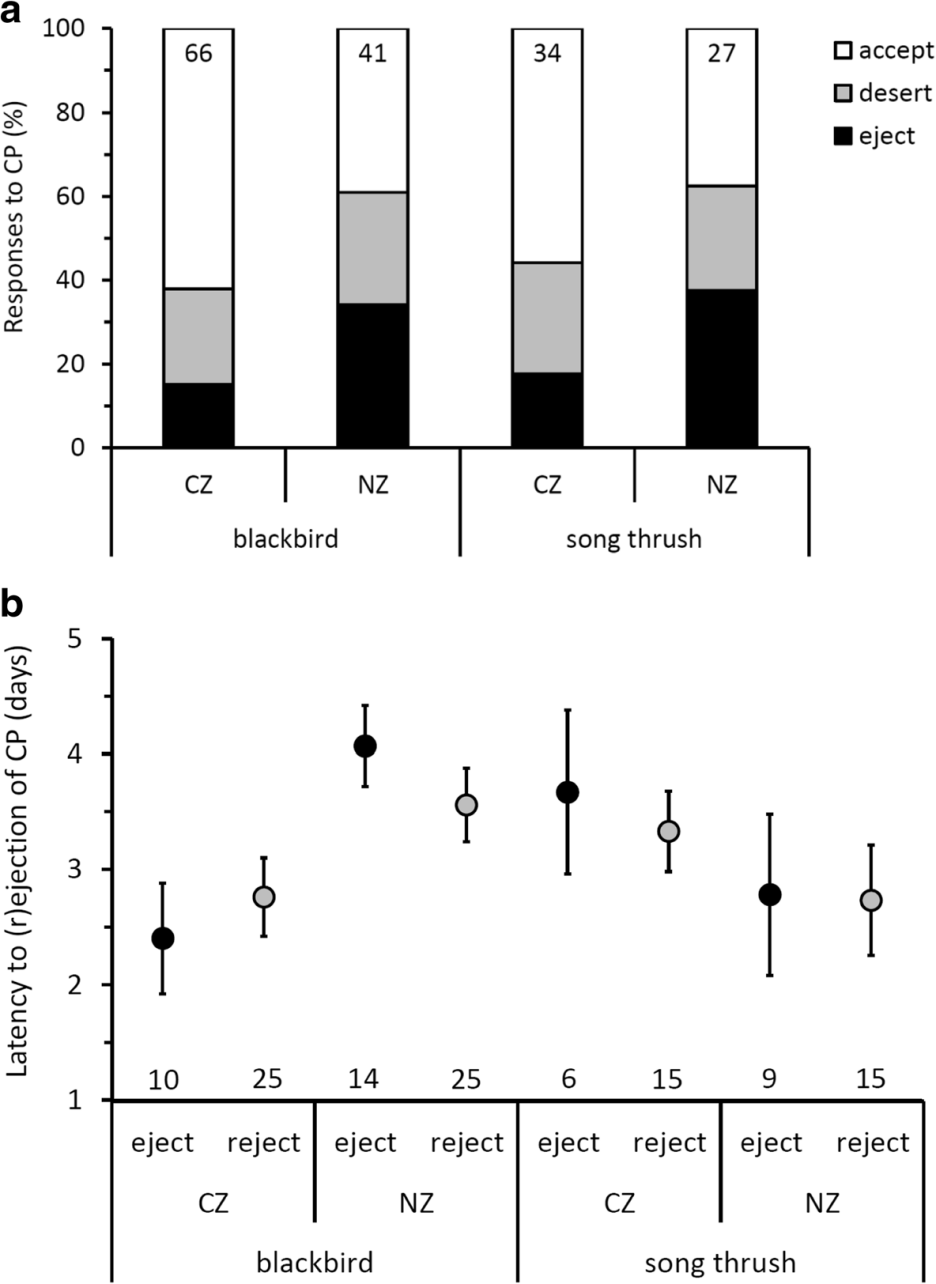
**Host responses to experimental conspecific brood parasitism (CP) measured as (a) egg rejection and (b) latency to egg rejection.** Responses to CP were compared between “CZ” (Czech Republic) and “NZ” (New Zealand) with low and high breeding densities, respectively; see hypotheses (ii) and (iii) in Introduction. Latencies to ejection (black) and to rejection (i.e., including desertion; grey) are presented as the raw data’s means ± SE. Sample sizes (nests) are given inside bars **(a)** or above x-axis **(b)**.

To test whether conspecific parasitism (CP) was responsible for the evolution of egg discrimination abilities in thrushes, we experimentally simulated conspecific brood parasitism by adding a real, natural egg from a different nest (Figure 1). Both blackbirds and song thrush rejected conspecific parasitic eggs at very high frequencies regardless of inclusion or exclusion of desertions (Figure 2a).

Both species rejected CP more often in areas with higher breeding densities, but the difference compared with areas with lower breeding densities was statistically significant only in blackbirds (Table 2, Figure 2a). No other predictors explained variation in CP rejection rates in any of the thrushes (Table 2).

**Table 2:** Egg rejection response and latency to rejection by thrushes

**Type of parasitism**	**Blackbird**			**Song thrush**		
	**ddf**	**F**	**P**	**ddf**	**F**	**P**
**CONSPECIFIC**						
**(R)ejection**						
Breeding density	**105**	**5.31**	**0.02**	41	3.01	0.09
Clutch	101	1.43	0.24	39	0.79	0.38
Nest stage	98	1.00	0.40	35	0.67	0.57
Laying date	97	0.92	0.34	38	0.63	0.43
**Latency to (r)ejection**						
Breeding density	48	2.94	0.09	11	1.22	0.29
Clutch	44	0.19	0.66	**11**	**5.13**	**0.04**
Nest stage	41	0.70	0.56	8	1.38	0.32
Laying date	47	0.64	0.43	7	0.05	0.82
**INTERSPECIFIC**						
**Ejection**						
Geography	**455**	**7.30**	**0.0008**	216	0.59	0.55
Egg model	**455**	**29.57**	**<0.0001**	**216**	**20.40**	**<0.0001**
G*E	446	1.11	0.33	206	0.24	0.79
Clutch	449	0.40	0.53	208	0.70	0.40
Nest stage	**455**	**5.41**	**0.001**	213	2.31	0.08
Laying date	448	0.04	0.85	212	1.63	0.20
**Latency to ejection**						
Geography	308	0.16	0.86	101	2.53	0.08
Egg model	**308**	**29.87**	**<0.0001**	101	0.33	0.57
G*E	300	2.53	0.08	95	1.33	0.27
Clutch	303	0.29	0.59	**101**	**7.99**	**0.006**
Nest stage	**308**	**3.86**	**0.01**	98	1.97	0.12
Laying date	302	0.05	0.82	97	0.40	0.53

**Figure 3:**
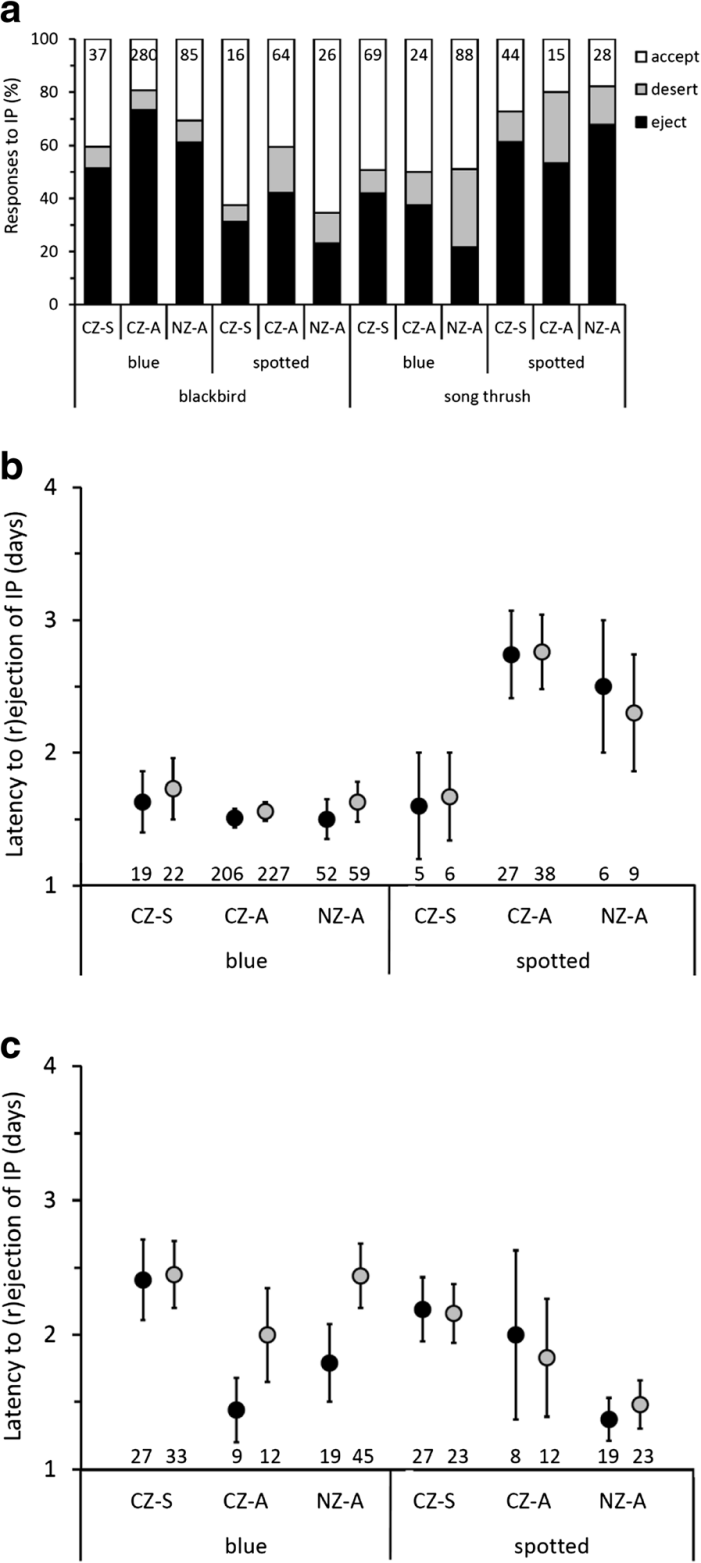
**Host responses to experimental interspecific brood parasitism (IP) with model cuckoo eggs measured as (a) egg rejection, and latency to egg rejection in (b) blackbirds and (c) song thrush.** IP was simulated by adding a blue or a spotted egg model (Figure 1). Responses were compared between areas in sympatry (CZ-S), micro-allopatry (CZ-A) and macro-allopatry (NZ-A) with common cuckoos (“CZ” – Czech Republic, “NZ” – New Zealand); see hypotheses (iv) and (v) in Introduction. Blackbirds **(b)** and song thrush **(c)** latencies to ejection (black) and to rejection (i.e., including desertion; grey) are presented as the raw data’s means ± SE. Sample sizes (nests) are given inside bars **(a)** or above x-axis **(b,c)**.

Response to conspecific egg in blackbirds includes nest desertion (i.e., abandoning the whole clutch), together with egg ejection (i.e., selective removal of a foreign egg), as rejection response to parasitism (see Results, and the Additional file 1: Appendices). All other responses (conspecific in song thrush, and interspecific in both thrushes) exclude desertion from analyses (for complete results, see Additional file 1: Appendix 2). Test statistics and P-values for non-significant terms are from backward elimination procedure just before the particular term (being the least significant) was removed from the model. Results of significant predictors from final models are in boldface. For effect sizes see Figures 2, 3 and Results. G = geography, E = egg model.

Blackbirds rejected conspecific eggs more quickly in areas of lower breeding density; the latency to rejection did not covary with breeding density in the song thrush (Table 2, Figure 2b). Song thrush latencies decreased with increasing clutch size: latency = 7.01(±1.96) + 1.10(±0.99) × Czech Republic - 1.12(±0.49) × clutch size). However, the sample sizes were quite small (range: 6–14 nests per treatment) for CP latency analyses, limiting our ability to conclude whether other factors may have covaried with latency.

### Responses to experimental interspecific parasitism vs. sympatry–allopatry

To test whether interspecific parasitism (IP) was responsible for the evolution of egg discrimination abilities in thrushes, we experimentally simulated cuckoo parasitism using artificial models resembling eggs laid by two widespread cuckoo’s host races (see Methods). Blackbirds and song thrush rejected the different model eggs at different rates: blue model eggs were rejected more often by blackbirds and spotted model eggs by song thrush (Table 2, Figure 3a).

Neither of the thrush species rejected experimental IP more often in areas sympatric with the cuckoo; the only statistically significant difference was a pattern opposite to what was predicted: we found higher rejection rate by blackbirds in micro-allopatry than in sympatry (Tukey–Kramer HSD: P = 0.02) and macro-allopatry (Tukey–Kramer HSD: P = 0.005), with sympatry and macro-allopatry being statistically similar (Tukey–Kramer HSD: P = 0.94; Table 2, Figure 3a). Rejection probabilities increased with advancing nest stage in blackbirds (Table 2).

Neither species rejected experimental IP faster in populations sympatric with the cuckoo (Table 2, Figure 3b, c). Latency to rejection decreased with advancing nest stage (in days) in blackbirds and decreasing clutch size in song thrush. Blackbirds rejected blue models faster than the spotted models (Figure 3b).

### Conspecific parasitism in the study populations

Estimated CP rates (see Methods) in Czech Republic blackbirds were 3.1% (n = 128 nests). This included two cases where two new eggs were laid per day and one nest where six eggs appeared in the nest within four days (i.e., two parasitism cases). We did not record any CP cases in New Zealand blackbirds. A putative case of CP was an extreme clutch of 8 eggs in a Czech Republic blackbird (only three nestlings hatched; median blackbird clutch size in the study population is four eggs [32]).

We did not observe any cases of CP in Czech Republic song thrush. Estimated CP rates in New Zealand song thrush were 2.2% (n = 90 nests). This included two eggs appearing in the nest within one day (total clutch was 5 eggs), and a new egg that appeared four days after clutch completion (original clutch of 3 eggs).

We further observed one case of Czech Republic blackbird clutch (two eggs) laid into a fresh song thrush nest, a probable case of nest usurpation (we do not know whether the blackbird removed any already laid song thrush eggs). Another Czech Republic blackbird female laid three eggs into an old song thrush nest (fledged earlier in the same year) but the nest was depredated before the end of incubation.

### Ejection costs/errors

We detected both rejection costs (own eggs damaged during successful rejection of a foreign experimental egg) and rejection errors (rejection of own eggs either with or without rejecting parasite egg). The latter may also represent rejection costs when damaged eggs were removed by nest owners before we checked the nest content. We found such events in both study species in areas of both sympatry and allopatry.

In blackbirds, frequency of costs/errors varied across populations and years from 2.8 to 10.0% (7 population-year-specific estimates with at least 10 nests per sample) with overall frequency of 5.6% (n = 323 nests). Frequency of costs/errors did not differ between Czech Republic and New Zealand populations (8.8% vs. 2.3%; Fisher's exact test P = 0.13). In song thrush, frequency of costs/errors varied across populations and years from 1.4 to 7.7% (4 population-year-specific estimates with at least 10 nests per sample) with overall frequency of 4.1% (n = 121 experimental nests). Frequency of costs/errors did not differ between Czech Republic and New Zealand populations (5.5% vs. 5.9%; Fisher's exact test P = 1.00). We also observed cases of possible rejection errors at control nests both in blackbirds (2.9%, n = 70 nests) and song thrush (6.9%, n = 43 nests).

## Discussion

The aim of our study was to solve an evolutionary and ecological paradox suggested by recent work [6]: if *Turdus* thrushes are unsuitable cuckoo hosts and could not coevolve with this interspecific parasite in the long term, why do they reject foreign eggs, including mimetic ones, and at high rates? Our data provide no consistent support for IP, and instead, strong directional support for CP as the main driver of the evolution of egg discrimination. This support was generally consistent across the two thrush species, populations, years and experimental treatments. Thus, the rejection of IP eggs by these passerine hosts is likely an epiphenomenon of selection by CP; this “collateral damage” hypothesis was previously considered but not supported for several other species known to reject foreign eggs [2,11,16,19-22]. This effect, documented so far only in a non-passerine waterfowl system [24], contributes critically to the evolutionary dynamics of realized unsuitability of potential hosts of IP because pre-existing defenses to reject foreign CP eggs also provides instantaneous protection from incipient IP (see [6]).

The single strongest evidence for the CP hypothesis here is that hosts selectively ejected (i.e., removed a foreign egg and incubated the rest of the clutch) foreign conspecific eggs at high frequencies (~20–40%), comparable to the rejection rates shown to some model cuckoo eggs in our experiments (Figures 2, 3). Some of these rates are exceptionally high, because published data show that, even strong rejecters of IP, often show nil rejection of experimental CP (e.g., [2]), including some other *Turdus* thrushes [22,62]. In other host taxa, where rejecters of IP reject some CP, they typically reject CP much less so than IP (e.g., [19,39,63]). Strikingly, these high rates of conspecific egg ejection were coupled with high nest desertion rates relative to control nests in blackbirds, confirming that nest desertion may be a specific response to CP in blackbirds (but not in song thrush) [60]. The implications of our findings for both thrush species are that they are poorly suited as potential hosts for both interspecific and conspecific parasites; the resulting low potential benefits of parasitizing conspecifics may explain the currently low levels of observed CP.

Both thrushes rejected simulated CP more often in populations of higher breeding densities as is predicted for conspecific parasites [43-45]. In turn, neither thrushes rejected simulated IP more often in populations with higher risks of cuckoo parasitism. This is contrary to predictions from general evolutionary theory [25,26] and empirical data from typical cuckoo hosts [29,49,50,54], but see [64]. Even in hosts of North American cowbirds, rejection rates are not higher in areas of allopatry than sympatry with IP [30].

Rejection costs and errors were similar in Czech Republic and New Zealand song thrush, and even smaller in New Zealand than Czech Republic blackbirds, contrary to general expectations that costs and errors should be more frequent where hosts have less reliable cues of IP presence [48]. Similarly in contrast to predictions of the IP hypothesis, rejection rates did not decrease and, in some cases, were even much larger in New Zealand than Czech Republic. In contrast, many other life-history parameters of introduced New Zealand blackbirds, including egg and clutch sizes, have changed predictably compared to native European populations [32]; this implies that IP (or its absence) is unlikely to have shaped behavioral and life history shifts in the introduced populations of these two thrush species.

Taken together, all these lines of evidence consistently support the view that thrushes evolved egg (r)ejection in response to CP, and reconciles divergent views on thrushes in the literature, which has previously classified them both as suitable [2] and unsuitable cuckoo hosts [5]. Both blackbirds and song thrush are striking outliers in many parasitism-related host traits in the context of IP. In contrast to known coevolved hosts of the cuckoo [54], thrushes do not show any consistent differences between areas of sympatry vs. allopatry with cuckoos in either aggression toward cuckoo dummies [6] or rejection response to model eggs [17; this study], including the repeatability of egg rejection and latencies to rejection [61]. In contrast to cuckoo hosts that strategically adjust their defenses against parasitism based on risks and costs [42], there is no such evidence in our study species [6,17,31,61]. Contrary to actual cuckoo hosts, thrushes are unable to raise the cuckoo chick successfully [6]. Together with our new findings here, the most parsimonious explanations for these divergent patterns is that thrushes are, or previously were, hosts impacted by conspecific parasitism.

Sophisticated egg discrimination has evolved in several species where effects of IP can be excluded [65,66] or played a secondary role to CP [9]; see also [11,16,24,63]. The costs of providing parental care for genetically unrelated young in CP are sufficient to drive evolution of some host defenses [67-69], even if CP occurs at low frequencies [70]. This is supported by the egg rejection abilities of hosts that are not parasitized by heterospecifics, e.g., in gulls [71], terns [72], murres [73], coots [68], rails [74], communally nesting cuckoos [69] and woodpeckers [75], and various passerines that are unsuitable cuckoo hosts, e.g., starlings (*Sturnus vulgaris*) [2], house sparrows (*Passer domesticus*) [65,66,70,76] and Eurasian tree sparrows (*P. montanus*) [77]. These patterns empirically reject theoretical arguments that CP is not sufficiently costly to select for host defenses.

Reports of CP are increasing in a number of species [10] and appear so disproportionately more in altricial birds [78]. CP was also detected in both blackbirds ([13]; K. Weidinger pers. comm.) and song thrush ([33], p. 1887 in [34]; K. Weidinger pers. comm.), and in the closely related redwings (*Turdus iliacus*; [22]), fieldfares (*T. pilaris*; [12]) and mistle thrush (*T. viscivorus*; [79]). Thus, CP might be relatively widespread in the genus *Turdus*. Here, we conservatively estimated conspecific parasitism rates as a ratio of cases of CP within a subset of nests found during laying stage and checked every day or every second day until the start of clutch incubation. This approach underestimates CP rates, see Materials and methods. Very high CP egg ejection rates (up to 40%) combined with high desertion rates of conspecific eggs (~20%, Figure 2) dramatically decrease the benefits of parasitizing conspecifics: 3 out of 4 parasite eggs would never hatch. Thus, the currently low potential fitness payoffs of parasitic strategy may explain the low rates of observed CP in these *Turdus* thrushes.

## Conclusions

Using consistent intra- and inter-continental methodology, we demonstrate that the rejection of non-mimetic egg models do not provide clear evidence for interspecific avian brood parasite-host coevolution. Fine scale discrimination, including of highly mimetic foreign eggs, may evolve due to selection pressures by conspecific parasites. Under the conspecific parasitism scenario, the rejection of model or real obligate brood parasitic eggs is effectively a by-product of adaptations related to conspecific parasitism. This “collateral damage” hypothesis provides a general framework that may explain why some potential victims of interspecific brood parasites remain un-exploited, despite their seeming suitability to serve as hosts. Therefore, future studies of egg discrimination should consider both interspecific and conspecific parasitism as viable explanations for the rejection of foreign eggs. Our study also highlights the utility of introduced avian populations as large-scale ecological experiments.

## Materials and methods

### Study model species

Both blackbirds and song thrush are common passerines; they breed in open woodlands and sub/urban areas, build conspicuous open nests, and may be available for parasitism by cuckoos both in space and time yet are currently rarely parasitized [6]. The blackbird lays eggs with a pale blue background and dense, fine, light-brown speckles (Figure 1). The song thrush lays a bright blue egg, with dark spots concentrated at the blunt egg pole (Figure 1).

### Study sites and species

*Sympatric populations*: We studied thrushes in Chválkovice (49°35'N, 17°17'E), Grygov (49°31'N, 17°18'E), and Lužice (48°51'N, 17°04'E) in the Czech Republic, Europe. These populations were in forested areas with cuckoos present during the breeding seasons (see [6]).

*Micro-allopatric populations*: We monitored thrushes in Olomouc (49°35' N, 17°15' E) and Brno (49°12' N, 16°38' E), also in the Czech Republic. These populations were in urban areas with cuckoos absent during the breeding seasons [6].

*Macro-allopatric populations*: We travelled to Auckland (36°51' S, 174°46' E), Hamilton (37°46' S, 175°16' E), and Tawharanui Regional Park (36°22' S, 174°49' E) on the North Island, New Zealand. Both blackbirds and song thrush were introduced to New Zealand in the late 19^th^ century [80] whereas (i) the common cuckoo was not [80], (ii) neither blackbirds nor song thrush act as regular hosts of native brood parasites in New Zealand [17,81], (iii) gene flow between New Zealand and Europe does not exist (both species are highly philopatric and non-migratory in New Zealand and New Zealand has a strict embargo on bird trafficking [82,83]), (iv) thrushes do not serve as cuckoo hosts in Asia [84] and there is no evidence for gene flow between Asia or introduced *Turdus* populations in Australia and New Zealand [34], (v) New Zealand populations breeding for ~150 years in isolation from cuckoos also show evolutionary changes in their breeding biology from their European source populations ([32] and references therein), and (vi) the biology of the New Zealand *Turdus* populations has not been affected by bottleneck effects [85]. Thus, we can be confident that our New Zealand populations did not experience any environmental cues [35], including visual [42] or acoustic signals of the cuckoo’s presence [86] for more than a century.

We conducted the experiments from April to July 2000–2011 in the Czech Republic and from September to November 2007–2009 in New Zealand. Because we could not work at all sites simultaneously even within the same country, we included first date of egg laying for each nest as a potential confounding variable in our models.

We avoided testing the same individual twice by working at each specific locality for only 2–3 weeks. We ringed some adults in 2008–2009 in New Zealand (n = 109) and 2008–2011 in Czech Republic (n = 154; [82]) which reliably allowed us not to test those individuals again.

We estimated parasitism rate as ratio of cases of CP within subset of nests found during laying stage and checked every day or every second day until the start of clutch incubation. This approach underestimates CP rates because (i) we did not conduct a genetic test of maternity of each freshly laid egg or completed clutch at our study site [78], (ii) most nests (72%) were not followed from the first egg laid, and (iii) ~25% of experimentally introduced conspecific eggs were ejected within one day (i.e., we could miss natural parasitic eggs which were ejected fast; see [78]).

### Egg experiments

We parasitized nests by introducing one of two types of artificial egg models or one natural conspecific egg (Figure 1). Egg models were made from polymer clay and painted with acrylic colors [87]. Size, mass and shape of artificial egg models were similar to real cuckoo eggs [31]. Host reactions toward egg models and real parasite eggs are similar [2,5,88]. “Blue model” (“redstart” type) is an immaculate pale blue and cuckoo-sized egg, representing a cuckoo gens which parasitizes the common redstart (*Phoenicurus phoenicurus*; [4]) in Europe. “Spotted model” (“meadow pipit” type) is a brown-grey egg, spotted with dark brown blotches, resembling the meadow pipit’s (*Anthus pratensis*) and its respective cuckoo host-race’s eggs [5]. We used these particular model types (i) because they were employed as a standard in many previous studies (including [6]), and (ii) it has been empirically documented that they are rejected at variable rates at our study sites [31]. This allows for meaningful comparisons between previous and our results.

Conspecific parasitism was simulated by adding a real, natural conspecific egg, collected from freshly abandoned clutches. Prior to use, each conspecific egg was checked for cracks that might also elicit egg rejection and only eggs without cracks were used. Each conspecific egg was used only once. All host eggs and conspecific experimental eggs were individually marked by water-proof, non-toxic marker on their blunt pole; eggshell numbering at the blunt pole does not affect cuckoo host responses to eggs [60,89].

Models painted with acrylic colors show different reflectances than natural conspecific eggs (Figure 1). This may be a problem for *interspecific* comparative studies (discussed by [57]) but not for the present work where comparisons are made *within* species. In fact, the low reflectance of artificial models, e.g. in UV part of the spectrum, is advantageous because it increases the avian perceived difference by these UV-sensitive hosts [90] between own eggs and a parasite model and, thus, provides a stronger test for host ability to reject non-mimetic eggs than a more “natural”, UV-reflective model might [91].

We checked the nest content daily, or every second day, for the standard 6-day period used in studies with European *Turdus* thrushes [2]. We classified host responses to experimental eggs as “ejected”, if the egg was missing whereas host eggs remained in the nest and were incubated, or “accepted”, if the experimental egg remained in an incubated clutch for at least 6 days.

Nest desertion in some *Turdus* thrushes may be a response to specific types of brood parasitism [33,60] or could result from other causes (human disturbance or inclement weather) at the nest. Therefore, we also inspected randomly chosen, unmanipulated, control nests for the 6-day period (these nests were handled just like experimental nests but no eggs were added).

We did not remove any host eggs because previous experiments showed that egg removal had no effect on the rejection behavior of several cuckoo hosts, including both of these thrushes [2,6]. Nonetheless, our experimental approach of adding (model) eggs rather than switching them with host own eggs might confound our results because clutch sizes are smaller in New Zealand than in Czech Republic [32]. Thus, the addition of a (model) egg would increase the relative clutch size and its visible area disproportionately more in New Zealand than in European nests. Therefore, we included the variable “clutch” in our models to test for such possible confounding effects.

### Statistical analyses

Overall, we obtained information on host responses under the 6-day response criterion for and for 685 blackbird nests and 372 song thrush nests. Although we included some already published data from Czech Republic ([6]; i.e., excluding data from other European countries) and New Zealand [31], the critical majority of the data reported here are new: 81% of blue, 80% of spotted and 100% of conspecific experimental data points had not been previously published. The inclusion of both old and new data also enabled us to test whether the relevant conclusions of [6] were simply an artifact of smaller sample sizes in that study.

We followed the statistical approaches of [6], and our results are therefore directly comparable with previous studies. We analyzed all data separately for the two thrushes. We were primarily interested in the effects of the type of experimental egg (prediction i), breeding densities (predictions ii and iii), and sympatry and allopatry with the cuckoo (predictions iv and v) on host behaviors, and we also controlled for factors that were shown to affect host discrimination behavior in some species in previous studies (see below). Rejection response was analyzed using generalized linear mixed models (binomial error and logit link function). Latency to egg rejection (in days) was analyzed with linear mixed models (identity link).

To test the CP predictions (ii and iii), our statistical models included, as the major predictor of interest, “breeding density” as a categorical predictor with two levels (low – Czech Republic vs. high – New Zealand). Breeding densities were similar and relatively low in the Czech Republic populations (both those classified as sympatric or micro-allopatric with the cuckoos) and statistically significantly lower than New Zealand breeding densities [32]. To test the IP predictions (iv and v) our statistical models included, as the major predictor of interest, “geography” as a categorical predictor with three levels (sympatry, micro-allopatry, macro-allopatry). Additional predictors included “egg model” type (blue vs. spotted) and its interaction with “geography”.

Initially, we fitted full models with these explanatory variables and “nest stage” (age of nest at start of experiment; categorical predictor with four levels: egg laying, 1–3 days of incubation, 4–9 days of incubation, 10 days of incubation to hatching), “laying date” (first egg laid; continuous predictor), “clutch” (clutch size at clutch completion; continuous predictor). Date was centered within each year for the Czech Republic and New Zealand separately to remove confounding effects of between-year variation of seasonal breeding and timing of experiments [32]. Random effects included population identity and year (entered as a nominal variable, [92]) to test for potential spatio-temporal correlation in the data.

We selected final models by backward elimination of non-significant terms. First, we sequentially examined the significance of covariates and kept a main factor of interest (CP: breeding density; IP: geography and egg model) in the model regardless its significance [92]. Reanalyzing the data with the full models does not change our main conclusions. In all models, the random effects (population identity and year) were very small (likelihood ratio tests; [93]), i.e., there was no significant temporal and population-specific variation in the data (presence/absence of random effects had no effect on our conclusions). The simpler models without the random effects, but with the same structure of fixed effects, had dramatically better fits (much lower AIC_c_) and very similar parameter estimates. Hence, we present results of the models without random effects [93].

All analyses were conducted in SAS v9.2 [94]. Results are shown as means ± SE unless stated otherwise.

## Competing interests

The authors declare that they have no competing interests.

## Authors’ contributions

All authors conceived and designed the study. PS performed the experiments in both the Czech Republic and New Zealand. TG and MEH helped with experiments in the Czech Republic and New Zealand, respectively. PS and TG analyzed the data and wrote the first draft. All authors edited drafts and approved the final manuscript.

## Supplementary Material

Additional file 1: Appendix 1 The statistical assessment and the resulting estimates of nest desertion as a response to brood parasitism in European blackbirds and song thrush. **Appendix 2.** Statistical analyses of egg rejection rates from models with nest desertion as a specific rejection response to parasitism excluded or included. Click here for file
