# Supplementary material for: Host responses to interspecific brood parasitism: a by-product of adaptations to conspecific parasitism?
Source: Front Zool. 2014 Apr 28;11:34. doi: 10.1186/1742-9994-11-34 (PMC4022367; doi:10.1186/1742-9994-11-34)
Supplement: Additional file 1: Appendix 1 — The statistical assessment and the resulting estimates of nest desertion as a response to brood parasitism in European blackbirds and song thrush. Appendix 2. Statistical analyses of egg rejection rates from models with nest desertion as a specific rejection response to parasitism excluded or included. [file 1742-9994-11-34-S1.doc]

**Host responses to interspecific brood parasitism: a by-product of adaptations to conspecific parasitism?**

Peter Samaš, Mark E. Hauber, Phillip Cassey and Tomáš Grim

**Appendices**

**Appendix 1 The statistical assessment and the resulting estimates of nest desertion as a response to brood parasitism** **in European blackbirds and song thrush.**

**Table A1.1** Statistical results from the model explaining nest desertion as a response (0 = nest survived the 6-day period, i.e. experimental egg accepted or ejected and control nests not deserted, 1 = nest deserted). Treatments = blue model, spotted model, conspecific egg, control. Test statistics and *P*-values for non-significant terms are from backward elimination procedure just before the particular term (being the least significant) was removed from the model. For *post-hoc* comparisons see Table A2. FEG = first egg laying date. For effect sizes see the main article text.

| **Predictors** |  | **Blackbird** |  | **Song thrush** | | |
| --- | --- | --- | --- | --- | --- | --- |
|  | **ddf** | **F** | **P** | **ddf** | **F** | **P** |
|  |  |  |  |  |  |  |
| Treatment | **669** | **5.32** | **0.001** | 355 | 0.88 | 0.45 |
| Breeding density | 669 | 0.64 | 0.53 | **355** | **3.27** | **0.04** |
| Clutch | 650 | 2.81 | 0.09 | 346 | 2.83 | 0.09 |
| Nest stage | 647 | 0.50 | 0.68 | 342 | 0.59 | 0.62 |
| Laying date | **669** | **12.32** | **0.0005** | 345 | 0.88 | 0.35 |

**Table A1.2** *Post-hoc* comparisons (Tukey HSD test) for “Treatment” levels of nest desertion between experimental and control treatment from final models (see Table A1.1).

| **Comparison** |  | **Blackbird** |  | **Song thrush** | | |
| --- | --- | --- | --- | --- | --- | --- |
|  | **df** | **t** | **P** | **df** | **t** | **P** |
|  |  |  |  |  |  |  |
| Blue vs. control | 669 | –0.54 | 0.59 | 355 | 0.21 | 1.00 |
| Spotted vs. control | 669 | 1.04 | 0.73 | 355 | –0.08 | 1.00 |
| Conspecific vs. control | **669** | **2.41** | **0.02** | 355 | 1.18 | 0.64 |

**Figure A1** Nest desertion rates (least square means with t-type 95% confidence intervals) of blackbirds and song thrush (a) with significant predictors (see Results) and (b) without predictors. When confidence intervals highly overlap between control nests and experimental nests within each species, the difference between treatments is non-significant. Numbers inside bars indicate sample sizes (n=nests).

(a)


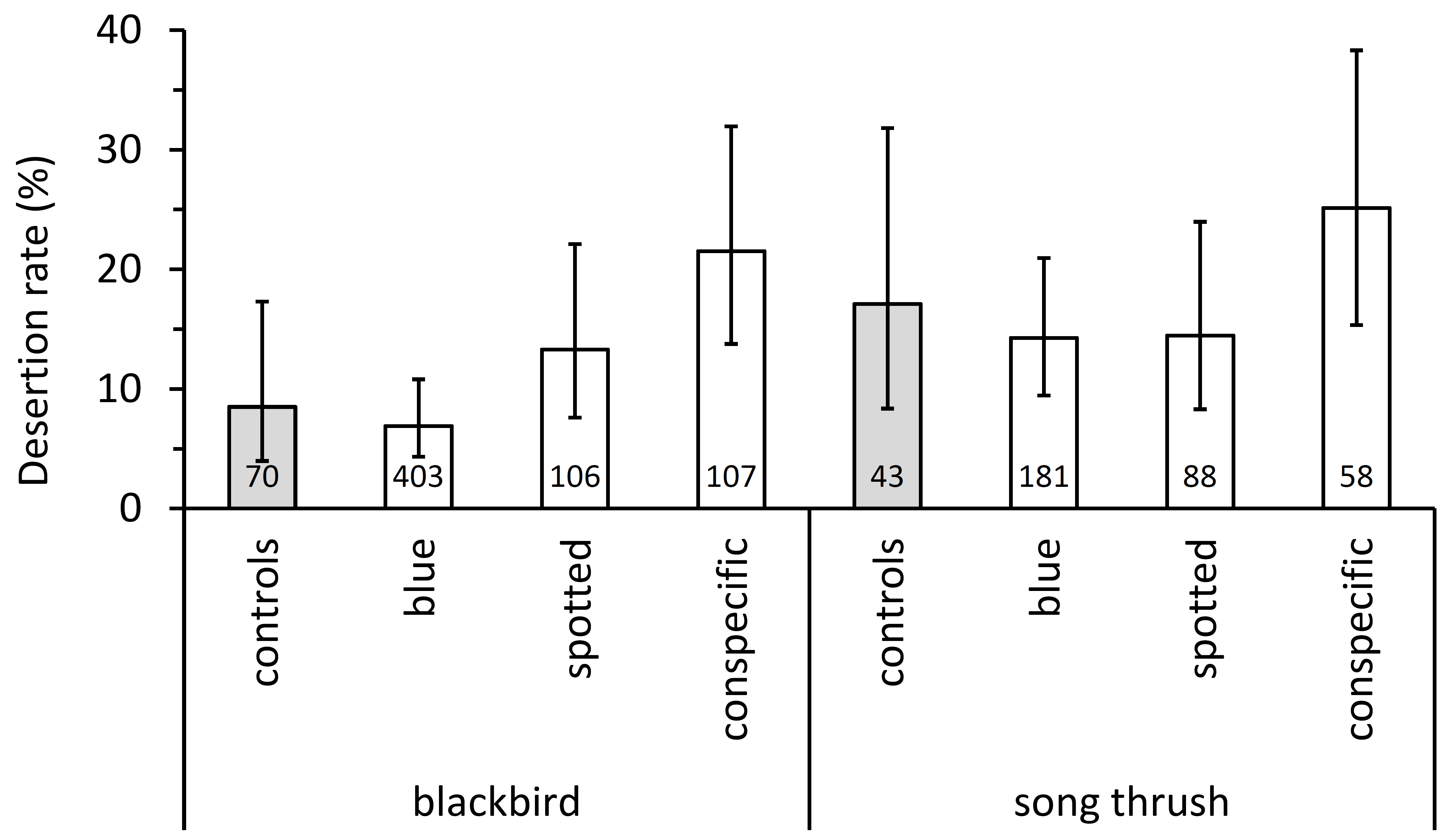


(b)


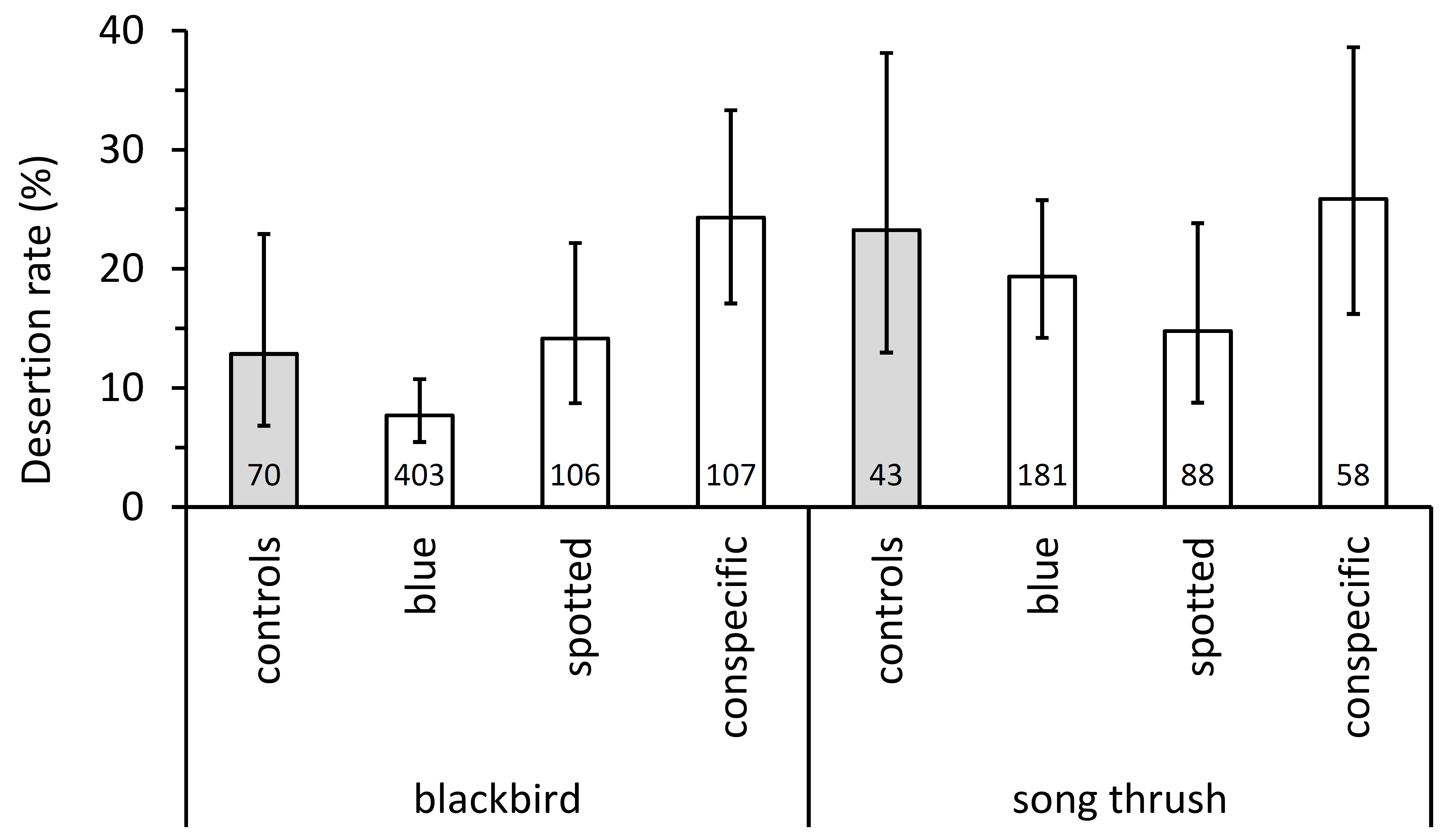


**Appendix 2 Statistical analyses of egg rejection rates from models with nest desertion as a specific rejection response to parasitism excluded or included.**

**Table A2.1** Egg ejection (desertion as response excluded) response and latency to ejection by thrushes. Test statistics and *P*-values for non-significant terms are from backward elimination procedure just before the particular term (being the least significant) was removed from the model. For effect sizes see the main article text.

| **Type of parasitism** | **Blackbird** | | | **Song thrush** | | |
| --- | --- | --- | --- | --- | --- | --- |
|  | **ddf** | **F** | **P** | **ddf** | **F** | **P** |
| **CONSPECIFIC** |  |  |  |  |  |  |
| **Ejection** |  |  |  |  |  |  |
| Breeding density | **79** | **6.32** | **0.01** | 41 | 3.01 | 0.09 |
| Clutch | 76 | 1.17 | 0.28 | 39 | 0.79 | 0.38 |
| Nest stage | 73 | 1.05 | 0.38 | 35 | 0.67 | 0.57 |
| Laying date | 72 | 0.24 | 0.62 | 38 | 0.63 | 0.43 |
|  |  |  |  |  |  |  |
| **Latency to ejection** |  |  |  |  |  |  |
| Breeding density | **22** | **8.27** | **0.009** | 11 | 1.22 | 0.29 |
| Clutch | 17 | 0.00 | 1.00 | **11** | **5.13** | **0.04** |
| Nest stage | 19 | 0.52 | 0.60 | 8 | 1.38 | 0.32 |
| Laying date | 21 | 0.21 | 0.65 | 7 | 0.05 | 0.82 |
|  |  |  |  |  |  |  |
| **INTERSPECIFIC** |  |  |  |  |  |  |
| **Ejection** |  |  |  |  |  |  |
| Geography | **455** | **7.30** | **0.0008** | 216 | 0.59 | 0.55 |
| Egg model | **455** | **29.57** | **<0.0001** | **216** | **20.40** | **<0.0001** |
| G*E | 446 | 1.11 | 0.33 | 206 | 0.24 | 0.79 |
| Clutch | 449 | 0.40 | 0.53 | 208 | 0.70 | 0.40 |
| Nest stage | **455** | **5.41** | **0.001** | 213 | 2.31 | 0.08 |
| Laying date | 448 | 0.04 | 0.85 | 212 | 1.63 | 0.20 |
|  |  |  |  |  |  |  |
| **Latency to ejection** |  |  |  |  |  |  |
| Geography | 308 | 0.16 | 0.86 | 101 | 2.53 | 0.08 |
| Egg model | **308** | **29.87** | **<0.0001** | 101 | 0.33 | 0.57 |
| G*E | 300 | 2.53 | 0.08 | 95 | 1.33 | 0.27 |
| Clutch | 303 | 0.29 | 0.59 | **101** | **7.99** | **0.006** |
| Nest stage | **308** | **3.86** | **0.01** | 98 | 1.97 | 0.12 |
| Laying date | 302 | 0.05 | 0.82 | 97 | 0.40 | 0.53 |

**Table A2.2** Egg rejection (ejection and desertion pooled) response and latency to rejection by thrushes. Test statistics and *P*-values for non-significant terms are from backward elimination procedure just before the particular term (being the least significant) was removed from the model. For effect sizes see the main article text.

| **Type of parasitism** | **Blackbird** | | | **Song thrush** | | |
| --- | --- | --- | --- | --- | --- | --- |
|  | **ddf** | **F** | **P** | **ddf** | **F** | **P** |
| **CONSPECIFIC** |  |  |  |  |  |  |
| **Rejection** |  |  |  |  |  |  |
| Breeding density | **105** | **5.31** | **0.02** | 56 | 1.88 | 0.18 |
| Clutch | 101 | 1.43 | 0.24 | 51 | 3.23 | 0.08 |
| Nest stage | 98 | 1.00 | 0.40 | 47 | 0.43 | 0.73 |
| Laying date | 97 | 0.92 | 0.34 | 50 | 1.33 | 0.25 |
|  |  |  |  |  |  |  |
| **Latency to rejection** |  |  |  |  |  |  |
| Breeding density | 48 | 2.94 | 0.09 | 23 | 2.01 | 0.17 |
| Clutch | 44 | 0.19 | 0.66 | **23** | **4.70** | **0.04** |
| Nest stage | 41 | 0.70 | 0.56 | 20 | 0.59 | 0.63 |
| Laying date | 47 | 0.64 | 0.43 | 19 | 0.04 | 0.85 |
|  |  |  |  |  |  |  |
| **INTERSPECIFIC** |  |  |  |  |  |  |
| **Rejection** |  |  |  |  |  |  |
| Geography | **501** | **7.51** | **0.0006** | 264 | 0.14 | 0.87 |
| Egg model | **501** | **24.46** | **<0.0001** | **264** | **16.03** | **<0.0001** |
| G*E | 485 | 0.86 | 0.42 | 253 | 0.09 | 0.92 |
| Clutch | 488 | 1.09 | 0.30 | 255 | 0.75 | 0.39 |
| Nest stage | **501** | **5.08** | **0.002** | 261 | 1.72 | 0.16 |
| Laying date | 487 | 0.18 | 0.67 | 260 | 1.56 | 0.21 |
|  |  |  |  |  |  |  |
| **Latency to rejection** |  |  |  |  |  |  |
| Geography | 357 | 0.08 | 0.93 | 145 | 0.35 | 0.70 |
| Egg model | **357** | **30.95** | **<0.0001** | **145** | **4.13** | **0.04** |
| G*E | 339 | 2.83 | 0.06 | 142 | 0.89 | 0.41 |
| Clutch | 342 | 0.03 | 0.85 | **145** | **5.37** | **0.02** |
| Nest stage | 354 | 2.06 | 0.10 | **145** | **2.72** | **0.05** |
| Laying date | 341 | 0.03 | 0.96 | 144 | 0.39 | 0.53 |
